# Phenomenological considerations on empathy and emotions in psychotherapy

**DOI:** 10.3389/fpsyg.2022.1000059

**Published:** 2022-10-10

**Authors:** Leonor Irarrázaval, Juan Pablo Kalawski

**Affiliations:** ^1^Facultad de Salud y Ciencias Sociales, Escuela de Psicología, Universidad de Las Américas, Viña del Mar, Chile; ^2^Unidad de Filosofía, Departamento de Psiquiatría y Salud Mental, Hospital Clínico, Universidad de Chile, Santiago, Chile; ^3^Facultad de Ciencias Sociales y Humanidades, Grupo de Investigación en Psicología Clínica y Psicoterapia, Carrera de Psicología, Universidad Autónoma de Chile, Temuco, Chile

**Keywords:** empathy, therapist’s empathy, client’s self-empathy, emotion schemes, existential meanings

## Abstract

In this article we will present a phenomenological approach to empathy and its relationship with emotions in the context of psychotherapy, highlighting the importance of empathy as a key element of the therapist-client relationship and therapeutic process, regardless of the therapist’s approach. We will use a consensus definition of empathy taken from phenomenologically oriented philosophy to analyze therapist’s empathy, as well as client’s self-empathy and client’s empathic communication with others. We will discuss emotions as they usually manifest in the context of psychotherapy, specifically describing how certain emotions can disturb empathic communication in close personal relationships and how it is possible to reestablish empathic communication in psychotherapy. This article it is not only based on evidence from scientific literature but also incorporates the authors’ practical knowledge of psychotherapy.

## Introduction

There are number of clinical studies and empirical findings that place empathy at the heart of psychotherapy. One of the first authors to highlight the crucial importance of empathy as a common factor of change in psychotherapy was Rogers (1957, 1963). Rogers suggested that when the therapist’s communicative style shows understanding and acceptance of the clients’ subjective experiences, this facilitates the client’s awareness and acceptance of their emotions. As a result, this process act as an antidote to the client’s feelings of worthlessness and helps them value their own subjective experience as a guide for their behavior. Rogers put empathy at the center of his approach, to the extent that he considered it one of the necessary and sufficient conditions for therapeutic change. Outside of the client-centered tradition, empathy has also been recognized as a crucial element in psychotherapy, for example in Kohut's (1977) psychoanalytic work. Empirical findings have suggested that therapists’ empathic responses toward clients’ subjective experiences help them to increase their awareness of emotions, recognize their needs, and develop more effective affect regulation strategies (Watson et al., 1998; Paivio and Laurent, 2001; Watson, 2002). Additionally, it has been suggested that therapist’s empathy helps clients develop positive self-treatment by accepting their subjective experiences as well as helps them feel more secure in close interpersonal relationships. Therapist’s empathy also helps clients overcome negative self-treatment, such as silencing, neglecting, oppressing, and controlling their subjective experiences, which can contribute to several interpersonal difficulties (Watson et al., 2014). Moreover, Barrett-Lennard (1997) argued that therapists’ empathy facilitates the development of self-empathy in clients, and that, as they become more empathic toward themselves, they become more open and empathic toward others.

Empathy has also been a central theme of philosophical analysis in the tradition of phenomenology. Since the end of the 19th century, when Vischer (1872) introduced the German term *Einfühlung* (from the Greek term empatheia: em “in” + pathos “feeling”), philosophical debates regarding its definition also began (Geiger, 1910). *Einfühlung* was translated into English by Titchener (1909) as “empathy” and introduced by Lipps (1907) into the field of the humanities and social sciences. The conceptual debate about empathy has continued until today involving other disciplines, such as cognitive sciences and psychology (Moran, 2004; Zahavi, 2022). This divergence regarding the definitions of empathy is problematic for empirical research as well as for training and intervention in psychotherapy. Therefore, we will use a consensual definition of empathy derived from phenomenologically oriented philosophy. Phenomenologists recognize that empathy allows access to the subjective experience of other people (including emotions) with the awareness of the other as “an-other” (Husserl, 1959; Stein, 1989).

Like empathy, emotions are also crucial to psychotherapy. Phenomenologists also agree that any form of conscious experience has a core affective dimension, even the simplest perceptual experience (Szanto and Landweer, 2020). Although emotions have always been present, academic focus on emotions as such is more recent than the focus on empathy. Early conceptualizations of psychotherapy viewed emotions as caused by unconscious drives. Thus, therapists were encouraged to work with these unconscious drives rather than with emotions *per se*. Currently, the dominant cognitive approach also views emotions as a byproduct, this time of thoughts. So, therapists are encouraged to work with thoughts, which is presumed to in turn change emotions. Even though this is still the mainstream view, in recent years there has been a movement toward recognizing that emotions are not just byproducts, but important processes crucial to change in psychotherapy (Lane et al., 2015). We will follow phenomenologists and psychotherapists who view emotion and cognition as forming integrated wholes (Colombetti, 2007; Greenberg and Goldman, 2019).

The emphasis on both empathy and emotions is clear in Emotion-Focused Therapy (Greenberg and Goldman, 2019), from which we will use the concept of “emotion schemes,” which are activated in the context of close personal relationships, such as couples, friends, families, and colleagues. We would like to highlight that these emotion schemes relate to “existential meanings” (Irarrázaval, 2022). Existential meanings are not related to problems of reason, that is, to metaphysical or philosophical questions, such as the question of freedom or the meaning of life. Existential meanings are related to a basic sense of oneself emerging in the interaction with other people that predispose us to communicate in a certain way toward ourselves, others, and the world. We will focus on disturbing emotion schemes, namely “primary maladaptive emotion schemes” (Greenberg and Goldman, 2019) or “core pain” (Timulak and Pascual-Leone, 2015) to describe how these emotion schemes can disturb empathic communication, as well as how to re-establish empathic communication in psychotherapy. This article it is not only based on evidence from scientific literature but also incorporates the authors’ practical knowledge of psychotherapy.

## Basic and extended empathy applied to psychotherapy

Within the tradition of European phenomenology there is agreement that, in its minimal definition, empathy is a mode of intentionality that makes it possible to access the subjective experience of another person, with the awareness of the other’s experience as being different from one’s own (Husserl, 1959; Stein, 1989). This minimal definition of empathy not only highlights the distinction between one’s own experience and that of an-other, but also focuses on the “foreign” experience of the other person, thus making it already possible to distinguish between empathy and a feeling of oneness. For instance, Allen’s (1976) reading of Husserl’s “*Das Kind. Die Erste Einfiihlung*” presents the developmental process through which the “first act of empathy” enables the child to recognize the other as an-other, with a life of their own and to view the surrounding world as an intersubjectively formed lifeworld. At an early stage of development, there is an instinctive relatedness between the child and their caregiver, which is based primarily on the child’s desire to have their needs fulfilled, so empathy – an intersubjective relatedness between two different individuals – is not yet developed. In the process of awakening to the surrounding world, empathy enables the child to recognize their caregiver as an individual with their own needs, which are different from the child’s. From this approach, empathy enables the distinction between one’s individual experience and that of another individual’s, so it is constitutive of intersubjectivity.

There is also agreement regarding a distinction between two forms of empathy: basic and extended empathy. Jaspers (1959) states that there are two different modes of understanding psychic phenomena, whose subjectively experienced character cannot be quantified or objectified in terms of scientific knowledge: the “static” and the “genetic” modes. The static mode involves the understanding of psychic states through the other person’s manifestations directly presented to us, including body movements, gestures, and facial expressions, as well as personal materials such as works of art and writings (objective psychopathology). Yet, it is important to bear in mind that this static mode of understanding is not a psychological understanding as such. Psychic or mental phenomena that are not directly present can only be psychologically understood by means of the genetic or empathic mode, making connections as to how one mental phenomenon emerges from another (subjective psychopathology). Accordingly, Jaspers’ static mode of understanding corresponds to a basic form of empathy, while his genetic or proper psychological mode of understanding corresponds to an extended form of empathy. Basic empathy enables direct access to the experience of others *via* perception of their non-verbal bodily expressions. Basic empathy is crucial in the mother-infant relationship, as the infant cannot communicate verbally (Winnicott, 1965). Basic empathy corresponds to what Gendlin (2012) called “the body’s relational knowing.” However, the experience of other persons includes a sense of themselves, others, and the world, which is not directly present in primary appearances of non-verbal bodily expressions. This is the starting point for extended empathic or psychological understanding (Irarrázaval, 2020). Extended empathy transcends apparently perceived phenomena, being basic empathy its condition of possibility. In other words, empathy can be extended to understand another person’s subjective experience from a psychological viewpoint (Jaspers, 1912, 1959), beyond one’s capacity to perceive their non-verbal bodily expressions.

From a phenomenological approach, both basic and extended empathy toward other people must preserve an awareness of the distinction between the experience of the empathizer and the experience of the empathized. Consequently, when referring to empathic or psychological understanding, we want to make a conceptual clarification with respect to other possible definitions of extended empathy. We are not conceptualizing empathic or psychological understanding as perspective-taking or cognitive empathy in the sense of an imaginative speculation of how one might feel if one were in another person’s situation (“putting oneself in the other person’s shoes”) or in terms of Fuchs (2017a) as an “explicit imaginary transposition into the other’s situation” (p. 43). In contrast, we conceptualize empathic or psychological understanding as a multilevel exploration of the unique experience of an-other, where this experience is different from one’s own. Ultimately, this psychological exploration is aimed at knowing the other person’s worldview, including not only affective-existential and cognitive aspects, but also cultural, social, and historical ones (Irarrázaval, 2020).

We do not want to suggest that theoretical inferences or imaginary simulations are not strategies that in some way can facilitate the empathic or psychological understanding of another person, but rather we want to point out that these strategies are not themselves empathy. In line with a precise phenomenological definition of empathy, the experience to be empathized with is not the experience of the empathizer, but the “foreign” experience of another person, namely the empathized (Zahavi, 2014a,b, 2015). Differently put, theoretical inferences or imaginary simulations focus on the experience of the empathizer who unilaterally uses their own experience to try to understand the experience of another person. Unilateral or “solipsistic anticipations” of other persons, such as interpretations, inferences, prejudices, and the like, imply that we somehow impute or project our own thoughts and imaginations onto the other person’s experience, without necessarily preserving the distinction between the experience of the empathizer and the experience of the empathized (Irarrázaval, 2020). The danger of losing this distinction is that the other person’s experience could be reduced to the experience of the one trying to understand it, eventually moving away from how the other person actually makes sense of their experience. This communicational mismatch can lead not only to confusion in the interaction with other people but even to psychopathology, for example, in extreme cases of paranoia in which the person attributes to other people intentions of persecution, harm and potential homicide, which are related to the condition of the person’s “ontological vulnerability” and not with the true intentions of others (Irarrázaval, 2022).

According to Zahavi (2016), empathic communication requires a form of second-person engagement between the empathizer and the empathized, namely a “second-person address.” This second-person engagement between “I” and “Thou” constitutes a new “we-identity” with a shared “communicational” project (“we-triadic structure”), which has the potential to transform our self-knowledge. On the one hand, the empathizer recognizes the experience of the empathized and, on the other hand, the empathized recognizes that their experience is being empathized with. When this psychological understanding is fully empathic, the person recognizes themselves as empathized with, which has been acknowledged as an important factor for change in psychotherapy (Rogers, 1957; Elliott et al., 2011; Watson et al., 2014). However, the communication between therapist and client differs from other types of close personal relationships, such as couples, friends, families, and colleagues, mainly because the conversation in psychotherapy focuses on the exploration of the client’s experience and not the therapist’s. The psychological understanding that guides the therapist is not motivated by mere curiosity or the spontaneity of an ordinary conversation of everyday life. Therapy does not consist of an exchange of experiences as occurs between members of close personal relationships, in the sense that space is usually given more or less equally for each to share their own experience. In psychotherapy it is only the client who shares their experience; the therapist can do it sometimes, but it is not the rule. In this way, the therapist is unilaterally oriented to psychologically understand the client’s experience through extended empathy, while the client is oriented, not to understand the therapist, but to understand themselves through self-empathy. In other words, there are “normative limits of mutuality” in the therapist-client relationship, in the sense that the therapist attempts to act on the client to psychologically understand their experience, which depends on a mutuality that is never to be complete (Buber, 1970).

Ratcliffe (2017) conceives therapist’s empathy as an extended exploratory process through which the client’s experience is progressively revealed to the therapist. How is the client’s experience revealed to the therapist? Hutto and Jurgens (2018) suggest that therapist’s empathy is extended in the sense that, interacting with the client, one is obviously moved by perceiving their bodily expressions and, most importantly, understands their situation by their stories. In this latter sense, therapist’s psychological or empathic understanding is conceived as a properly discursive and, especially, narrative-driven form of engagement with the client. Precisely because the therapist is oriented toward psychological or empathic understanding of the client and not *vice-versa*, the therapist’s interventions must be in accordance with the client’s experience, not what the therapist thinks that experience is, nor the therapist’s experience about the client’s experience. Although the experience of the clinician in interaction with the client has been pointed out in its possible contribution to the psychiatric diagnosis, for example in schizophrenia (Rümke, 1990), in psychotherapy the therapist’s awareness of their own experience emerging in the interaction with the client serves to preserve the required distinction between them, so that the therapist’s intervention responds to the actual experience of the client that is being revealed in the therapeutic process. Thus, the therapist does not unilaterally attribute mental states to the client, such as beliefs, intentions, meanings, and emotions, nor does the therapist focus on imaginatively simulating how they would feel in the client’s situation. The therapist is also not a passive observer but is in a face to face “second-person relationship” (León et al., 2022), oriented to psychologically or empathically understanding the client through their displaying or revealing their subjective experience. This implies that the depth of the therapist’s empathy will relate to the depth of the client’s communication (Gendlin, 1986).

When the client feels understood, that is, feels empathized with by the therapist, a full empathic communication is achieved, facilitating change in the psychotherapeutic process. Additionally, as the therapist psychologically or empathically understands the client’s experience, the client empathically understands their own experience through self-empathy. Making a person’s own experience the focus of their empathic self-understanding is indeed the focus of psychotherapy. On many occasions, people do not understand their own experience. For example, they experience emotions that they consider inappropriate to a situation, or they act in ways that are incomprehensible to themselves because they are dissociated or disconnected from their own subjective experience. Of course, self-empathy does not require preserving the distinction between the experience of the empathizer and the experience of the empathized, since in this case empathizer and empathized are the same person. What this case requires is the distinction between pre-reflective and reflective levels of self-awareness which are processes of selfhood of the first-person perspective (Zahavi, 2020). So, in self-empathy, the distinction between pre-reflective and reflective self-awareness takes the place of the distinction between first- and second-person perspectives in empathy between different people, for instance, therapist and client. This relationship between pre-reflective and reflective levels of self-awareness from the first-person perspective is crucial to make explicit in psychotherapy for all kinds of concerns, from mild relationship conflicts or vocational issues to severe difficulties such as paranoid thoughts or hallucinations. In psychotherapy, the relationship between pre-reflective and reflective levels of self-awareness is addressed through self-empathy, which is an explicit psychological understanding of the client’s first-person experience facilitated by the therapist’s extended empathy. In other words, self-empathy involves the application of reflective awareness to the client’s own pre-reflective experience. Self-empathy is thus an extended, narrative form of empathy. The importance of applying reflective awareness to one’s own experience is widely regarded as crucial to mental health, from psychoanalysis’s dictum of making the unconscious conscious, to the Rogerian view of congruence between experience and self-concept, to the cognitive-behavioral task of becoming aware of one’s own cognitive distortions. Some readers may thus wonder whether self-empathy is tantamount to insight. As with the word “empathy,” the word “insight” can mean different things. In the psychotherapy context, the term often refers to an event in which the person makes new connections (Hill et al., 2007). Under this meaning of the word, insight is a product of self-empathy. We would like to highlight that self-empathy is a process rather than a state or an outcome.

## Emotion schemes and maladaptive emotion schemes or core pain

One of the tensions in the conceptualization of emotions relates to which aspect of emotions is emphasized. On the one hand, emotions are internally felt. On the other, emotions are intentional, that is, about the world. We believe that Müller’s (2022) proposal is a promising way to integrate these two aspects into a coherent phenomenology of emotions. He argues that the way one feels in having an emotion is not a perception-like awareness of “evaluative properties” of its object, but instead constitutes the taking of a stand or position about this object considering its evaluative properties. Many therapists teach their clients that they can change their feelings by changing their thoughts. To some extent, this is true, but it is also true that, many times, changes in thoughts do not lead to changes in emotions. In addition, Colombetti (2007) has argued that it is phenomenologically implausible for cognitive appraisals to precede emotions. She proposed that appraisal is fully integrated into emotional experiences. Emotions, thoughts, action, and interaction form integrated wholes. In the psychotherapy literature, Greenberg and his colleagues (Greenberg et al., 1993; Greenberg and Paivio, 1997; Elliott et al., 2004; Greenberg and Goldman, 2019) have proposed the concept of “emotion scheme” to refer to these integrated wholes. The word “emotion” in emotion scheme highlights that emotions play a central role in these multicomponent organizations. Emotion schemes are learned through experience and produce complex, idiosyncratic emotional experiences when they become activated. Emotion schemes include five elements. The central, organizing element in an emotion scheme is the emotion itself, such as anger, fear, or tenderness. The perceptual element is composed of the cues that activate the emotion. For example, a client recalled the way everybody looked at his mother when she was drunk at a party. Emotion schemes also have a bodily element, including such experiences as clenched jaws, fast breathing, etc. The fourth element are existential meanings, that is, what it means for oneself to exist in the world when the emotion scheme is active. These meanings are based on the emotion but also include idiosyncratic elements. For example, fear is associated with danger, but a person may symbolize a specific experience as “I felt like the Little Red Riding Hood walking alone in the forest.” The fifth element of emotion schemes is the motivational element. This latter element includes action tendencies, such as wanting to hide or wanting to punch somebody. The motivational element also includes a deeper aspect: existential needs, such as the need for validation and the need for support.

Emotion schemes are not exclusive to psychopathology. Rather, they are a general concept to explain psychological functioning. Additionally, emotion schemes not only include emotions in a narrow sense of just labels such as “angry” or “sad.” Emotions are more than mere labels for subjective feelings. They always imply a positioning in the world (Müller, 2022). To describe difficulties with empathy in mental health concerns, we focus on disturbing emotion schemes, namely “primary maladaptive emotion schemes” (Greenberg and Goldman, 2019) or “core pain” (Timulak and Pascual-Leone, 2015). Greenberg and Goldman (2019) explain the concept thusly:

Primary maladaptive emotions are core painful emotions that are more a response to past unresolved issues and unmet needs (based often on traumatic learning) than an adaptive response to current circumstances. Consequently, they do not prepare the individual for adaptive action in the world. Maladaptive primary feelings are responses that may once have served a useful purpose, but when presently activated in current situations they lead to responses that are now inappropriate (e.g., fear in response to affection from a past abuser is now activated in response to a loving other or feeling the shame of inadequacy when one is criticized, which stems from invalidation by one’s peers or parents). As such, they do not provide useful information to guide present action for the adult. Because maladaptive primary emotions do not change with changing circumstances, they often leave the individual experiencing them feeling stuck, hopeless, and helpless (i.e., depressed or anxious). Present functioning is ruled by the past, and the newness and richness of the present moment is lost (p. 65).

As is implied by the above definition, primary maladaptive emotion schemes can blind the person, rendering them unable to visualize the other’s experience, as the current interaction is filtered through the lens of past painful experiences. Specifically, then, we propose that primary maladaptive emotion schemes disrupt basic empathy. Therefore, these schemes not only lead to unpleasant emotions; they also lead to the interpersonal difficulties integral to mental health concerns. These interpersonal difficulties, in turn, lead to more psychological pain. We should clarify that we are not claiming that maladaptive emotion schemes are the only possible disruptor of basic empathy. For example, Robinson et al. (2021) suggested that difficulty with basic empathy can be due to a mismatch between neurotypical and neurodivergent forms of intersubjectivity. Nevertheless, we suggest that primary maladaptive emotion schemes are a common impeder of basic empathy. Timulak and Pascual-Leone (2015) articulated that these maladaptive, core emotions can be clustered as sadness-related, shame-related, and fear-related. Let us briefly review each of them:

- Shame: Shame is basically an adaptive emotion, as it alerts the person that their social standing is in peril (Sznycer et al., 2018). This emotion thus motivates one to change one’s behavior to restore harmonious social relations. There is, however, a difference between shame as a response to a specific situation and internalized shame as a core sense of oneself. This primary maladaptive shame involves a sense of worthlessness of the whole person. This shame often comes from a history of humiliation and is part of a core sense of self. For people with primary maladaptive shame, any self-expression is automatically accompanied by a feeling of shame (Greenberg and Paivio, 1997). Maladaptive shame thus inhibits authenticity and disrupts interpersonal flow. The person is not attuned to the actual interaction but instead assumes that the other holds a negative view toward themselves. From the perspective of the other, the behavior of the person with maladaptive shame is not transparent, as it cannot be understood based on the ongoing interaction.
- Sadness: This emotion is associated with isolation and loss of connection with other people. It points to the existential need for closeness and love – more specifically, being able to count on someone for comfort, protection, support, and, generally, help. This is a healthy need and sadness is basically a healthy emotion. When a child is emotionally neglected, they build a maladaptive sadness scheme, characterized by a tendency to view situations as abandonment. For example, a friend being late to lunch might be taken personally and become emotionally hurtful. The primary emotional response in these situations is sadness, but the person will often defend against this experience and either lash out at the friend or adopt a posture of resignation. As with the case of maladaptive shame, maladaptive sadness clouds interpersonal attunement. In the case of the friend being late, there are multiple possible reasons for this, and a healthy response would involve being open to contextual information. But the person with a maladaptive sadness scheme will instead make an abandonment interpretation that may not be consistent with the situation. This impedes an empathic attunement to the friend’s experience.
- Fear: This is a crucial emotion that mobilizes organisms to escape from danger. From an evolutionary standpoint, mistaking a dangerous situation as safe is deadly. However, mistaking a safe situation as dangerous may be costly, but preferable to the alternative. Thus, it makes sense that people respond with fear when faced with ambiguous situations. This natural tendency is even more pronounced when people have experienced significant trauma during childhood. A maladaptive fear scheme then leaves people vulnerable to experiences of terror and dissociation in response to situations in which there is a potential for loss of control (Timulak and Keogh, 2020). It is important here to emphasize that primary maladaptive fear is different from the more common kind of anxiety that is produced by thoughts. For example, thinking about doing poorly on a job interview can generate anxiety. By contrast, primary maladaptive fear is automatic and pre-reflective. When the person is in the throes of terror, attention becomes narrowly focused on escaping the situation, impeding the “decentering” necessary for empathy (Fuchs, 2017b).

## Empathy and mental health problems

Empathy issues have already been noted in dark personality traits, as well as in autism spectrum disorders, for example (Irarrázaval, 2020). Here, however, we suggest that empathy issues are common in a wide range of mental health concerns. Rather than addressing specific mental health concerns, we will discuss in general terms their relationship with empathy. First, we first need to briefly explain the two main sources of mental health concerns proposed by Greenberg et al. (1993). According to these authors, psychological dysfunction stems from: (1) the ongoing conscious construction of meaning and (2) the automatic activation of painful pre-reflective experience. Conscious meaning is the application of socially constructed symbols to make sense of one’s experience. As humanistic authors (Rogers, 1961; DeRobertis, 2006) have pointed out, these constructions can conflict with organismic (pre-reflective) experience, as in the statement “boys do not cry.” Someone who says this to a child is most likely not being empathic. Not receiving proper empathic or psychological understanding then affects self-empathy, as in coming to believe that boys do not cry and trying to apply that generalization to one’s own experience. To illustrate the second source of dysfunction, let us take the example of a person who is very sensitive to abandonment. Being very sensitive to abandonment involves the automatic activation of painful experience. This experience is pre-symbolic, embodied, and not representational (Gendlin, 1982). In addition to agreeing with Greenberg et al. (1993) that pre-reflective experience can itself lead to mental health concerns, we note that this experience is not only subjective but also intersubjective and thus tied to difficulties with empathy toward others. This is clear, for example, in the sensitivity to abandonment, an inherently intersubjective experience.

Clinical experience shows that mental health concerns always feature interpersonal difficulties. In fact, complaints such as “I am depressed” or “*estoy enferma de los nervios*” may be an artifact of the ubiquity of cultural messages that encourage the internalizing of problems. In addition, the exploration of symptoms quickly runs out. By contrast, exploration of interpersonal concerns is richer and feels alive. Some clients skip the symptom talk altogether and simply complain of interpersonal difficulties in the context of close personal relationships, such as couples, friends, families, and colleagues. Such clients often meet the requirements for psychiatric diagnoses, but the symptoms are not what motivate them to seek help. In the present article, we do not pursue to explore the specific mechanisms that may lead to specific kinds of interpersonal difficulties. Rather, we would like to take a general look at interpersonal difficulties considering our previous discussion of empathy. As we saw, it is useful to distinguish between basic and extended empathy. We would like to propose that the interpersonal difficulties present in mental health concerns involve difficulties with basic empathy. That is, what is disturbed is the spontaneous intersubjective flow necessary for social functioning.

Emotion schemes that interfere with basic empathy are those in which a self-disturbing experience of a previous life situation is pre-reflexively reactivated. These emotion schemes can disturb an empathic response. This means that, in certain situations, a person’s emotional reaction is tied to a previous disturbing experience and does not correspond to the subjectivity of another person in the present. For example, a depressed person may incorrectly assume that others do not like them, or a person with post-traumatic difficulties may interpret benign behaviors from others as rejecting. It is worth pointing out that the disturbing emotion schemes are unique and personal in terms of existential meanings, so certain situations that could be seen as obviously disturbing or even traumatic do not necessarily entail disturbing or traumatic consequences in the person who experienced them. Conversely, certain situations that could be seen as obviously not disturbing or even trivial, can have disturbing or traumatic implications. For example, a person who suffered bullying in childhood, when exposed to a harmless joke might not have an emotional reaction of humor, but of shame or anger, being unable to take the joke as such, but consider it offensive due to previous disturbing or traumatic experiences. However, this empathic disturbance would not occur in other situations of everyday life that are not related to the previous disturbing or traumatic situation, which the same person could respond empathetically to.

## Using extended empathy to reestablish basic empathy

In psychotherapy, psychological or empathic understanding is acquired through the unfolding of disturbing emotion schemes involved in the client’s subjective experience. This unfolding includes emotional expression on the part of the client. Additionally, the client discloses the existential meanings that challenged their vulnerabilities in the context of a close personal relationship. The therapist promotes self-empathy in the client, with the corresponding distinction between aspects of their emotional schemes that relate to original disturbing or traumatic experiences. In this manner, the client begins to distinguish between those emotional reactions that have to do with a past life situation from those emotional reactions that correspond to the present life situation. Thus, interaction with the therapist enables the client to reestablish empathic communication through dialog, overcoming the client’s solipsism. Here, solipsism refers to a “self-centered” state (Irarrázaval, 2018) in which the other person’s point of view is reduced to one’s own. In other words, blinded by their own emotional disturbances, the person cannot see the other as an-other, independently from their own emotional experience. In such solipsistic state, a person projects their own experience onto the other’s experience, manifesting an empathic failure.

For example, a client who experienced sadness without knowing its true origin, initially attributed it to an increased work stress because of the pandemic. In this situation there was a difficulty with self-empathy. In psychotherapy, the therapist’s extended empathy and the client’s self-empathy allowed for the exploration of the experience of sadness and its existential meaning. In this way, the client understood the existential meaning of abandonment connected to the recent loss of a loved one, a grief that had been blocked. The emotional expression of sadness and its corresponding association with the loss situation resulted in symptomatic relief and disclosure of the existential need for closeness, which moved the client from isolation to actively seeking social contact. In another scenario, a woman may communicate in an aggressive, demanding or dominating way with her husband, impeding empathic communication between the two. Aggressive, demanding, or dominating interactions are different from assertively expressing one’s needs. In the latter case, the person is aware that their needs are their own, thus preserving the self-other distinction necessary for empathy. If the woman is aggressive, demanding, or dominating with her husband, she addresses him as the target of her anger without visualizing his subjectivity. Also, in a close personal relationship, assertive communication usually occurs automatically as part of the interactional flow. Thus, aggressive, demanding, or dominating interactions in close personal relationships usually involve difficulties with basic empathy. In this case, the therapist deploys extended empathy to unfold the woman’s experience. It may then become clear to both the therapist and the woman that anger is not her primary feeling. Rather, there is a core feeling of loneliness and sadness. This sadness, once fully experienced, points to the need for support. By recognizing this need, the woman has moved from seeing her husband as a target of anger to seeing him as a potential source of support. This allows her to be more open to visualizing his subjectivity. Next, the woman can express to her partner her need for support in a non-blaming manner. This move makes the woman more transparent to her husband, which makes it easier for him to empathize with her and allows him to respond compassionately, meeting her need for support. Over time, as partners respond positively to each other’s existential needs, each partner’s disturbing emotion schemes transform, which helps restore basic empathy to the relationship. Additionally, Kalawski (1997) reported another interesting example of the relationship between emotions and empathy. A client expressed resentment toward her partner. The therapist then guided her through an exercise consisting of adopting the breathing, posture and facial expression of a person experiencing tenderness. After this exercise, the client said she spontaneously shifted her view of the situation and was able to consider her partner’s experience. The client shifted her literal and her emotional positioning (Müller, 2022), facilitating basic empathy. Recent studies have also shown that the emotion of tenderness facilitates the process of couple therapy (Veach, 2016; McNally, 2020). We believe that tenderness as an emotion may be at the core of empathy and suspect that maladaptive emotions and tenderness mutually inhibit each other. Thus, at times, working directly with tenderness can help improve basic empathy, while at other times it may be more helpful to directly address whatever maladaptive emotions may be present.

## Conclusion

In this article, we have addressed the relationship between empathy and emotions, with an application to psychotherapy. We have employed a basic as well as an extended notion of empathy as defined in the philosophical phenomenological tradition. We have also included the notion of self-empathy. We have proposed that an extended form of empathy is the one employed by the therapist and that self-empathy is the form developed by the client in psychotherapy. Regarding emotions, we have employed the notion of emotion schemes, and proposed that maladaptive or disturbing emotion schemes impede basic empathy. We have argued that certain life situations negatively affect people’s basic empathy through maladaptive emotion schemes and that the therapist’s extended empathy can reestablish it. In this sense, therapist’s extended empathy can be conceived of as an external event that facilitates processes of change in psychotherapy. We have also suggested that therapist’s extended empathy develops client’s self-empathy in psychotherapy. Self-empathy improves client’s narrative coherence. Studies have shown that this improved narrative understanding of disturbing emotion schemes is associated with psychotherapy outcome (e.g., Krause, 2005; Basto et al., 2021). This self-understanding in turn leads to a reestablishment of client’s basic empathy and facilitates their extended empathy toward other persons, preserving the distinction between client’s and others’ experiences. We believe that our conceptualizations of basic and extended empathy and understanding of their relationship with emotions may shed light on the common factors of the therapeutic process by specifying the ways in which therapist’s extended empathy and client’s self-empathy are put into play. This has significant implications for future empirical uptake of the notion of empathy within phenomenological research, as well as for use in education and to advance psychological interventions beyond a specific psychological theory or model.

## Data availability statement

The original contributions presented in the study are included in the article/supplementary material, further inquiries can be directed to the corresponding author.

## Author contributions

All authors listed have made a substantial, direct, and intellectual contribution to the work and approved it for publication.

## Funding

LI was supported by the Chilean National Research and Development Agency: “ANID/FONDECYT de Iniciación/2020/FOLIO 11200138.” Open access publication fee was funded by “Programa de Apoyo al Acceso Abierto,” Dirección de Investigación, Vicerrectoría de Investigación, Universidad de Las Américas, Chile.

## Conflict of interest

The authors declare that the research was conducted in the absence of any commercial or financial relationships that could be construed as a potential conflict of interest.

## Publisher’s note

All claims expressed in this article are solely those of the authors and do not necessarily represent those of their affiliated organizations, or those of the publisher, the editors and the reviewers. Any product that may be evaluated in this article, or claim that may be made by its manufacturer, is not guaranteed or endorsed by the publisher.
